# Hypericin Enhances Paclitaxel-Induced B16-F10 Cell Apoptosis by Activating a Cytochrome *c* Release–Dependent Pathway

**DOI:** 10.3389/fphar.2021.652452

**Published:** 2021-08-04

**Authors:** Liyun Sun, Zixuan Li, Huoli Shang, Xiujuan Xin

**Affiliations:** East China University of Science and Technology, Shanghai, China

**Keywords:** hypericin, paclitaxel, apoptosis, B16-F10 cells, mitochondrial dysfunction, cytochrome c release

## Abstract

The enhanced inhibitory effect of paclitaxel (PTX) combined with hypericin (HY) on B16-F10 cells may be realized through the ROS-related cytochrome c release pathway. The apoptotic characteristics of the B16-F10 cells, such as DNA fragmentation, chromatin condensation, and apoptotic body formation, were all enhanced in the combined treatment group. Further investigation showed that the combination of paclitaxel and HY could increase the level of mitochondrial damage and the concentration of cytochrome *c*, causing the expression of caspase-3 and the cleavage of PARP.^1^. Compared with paclitaxel or HY alone, the level of reactive oxygen species (ROS) increased significantly, while glutathione reductase (GR) activity and intracellular glutathione (GSH) levels decreased significantly in the combination group.

## Introduction

Skin melanoma is one of the top three most malignant tumor types because of its propensity to metastasize and insensitivity to traditional clinical treatment strategies, such as radiotherapy and chemotherapy (Teicher, 2002). Currently, surgery is still the preferred therapy for many patients with malignant melanoma, and prognosis remains poor (Rager et al., 2005). Therefore, there is an urgent need to identify effective drugs for malignant melanoma therapy. The B16-F10 cell line is a mouse tissue melanoma cell, which is frequently used as a model of melanoma for drug screening (Nanni et al., 1983).

Paclitaxel has been successfully employed as a broad-spectrum anticancer drug in clinical applications. This drug binds to the microtubule to stabilize the microtubule structure of the cells, blocking the cell cycle at the G2/M phase (Clemente et al., 2019). There are reports that paclitaxel is effective against metastatic breast cancer, ovarian cancer, non–small-cell lung cancer, melanoma, and other cancer types (Kalechman et al., 2000; Yao et al., 2007; Meng et al., 2017). However, the use of paclitaxel in cancer treatment has reached a bottleneck in multidrug resistance. It has been reported that paclitaxel can form a complex with GSH, subsequently being effluxed outside or desensitized by inducing the expression of resistant proteins, such as P-gp, MRP_2_, or MRP1 (Versantyoort et al., 1993; Henness et al., 2002; Brown et al., 2004; Padowski and Pollack, 2010). Dose increase is a common clinical strategy for receding drug resistance, but high-dose delivery induces undesirable toxicity in patients (Alexiades, 2006).

Combination with other drugs at a low dose has been employed to avoid the severe side effects of high-dose paclitaxel in patients. Eliminating intracellular antioxidant capacity might be effective at strengthening paclitaxel therapy efficiency in the clinic, such as reducing ROS and reduced-state glutathione (GSH) levels. Mitochondria-targeting GSH-sensitive drug delivery platforms have recently been suggested to strengthen the effect of chemotherapy.

Hypericin (HY) is a phytochrome extracted from *Hypercom perforatum* which acts as a photosensitizer with specific photochemical properties (Rosa and Bentley, 2000). In recent years, photodynamic therapy (PDT) has attracted the attention of researchers as a novel active cancer treatment strategy (Pace and Mackinney, 1941; Thomas et al., 2010). Hypericin can be photoactivated under UV light and generates a high dose of reactive singlet oxygen molecules (Hadjur et al., 2010), which induces lipid peroxidation, enhances superoxide dismutase activity, and decreases cellular glutathione levels and photohemolysis of red blood cells (Vandenbogaerde et al., 1998). Due to its biological activity in the induction of apoptosis in cancer cells, hypericin is considered a new anticancer drug for PDT (D'Hallewin et al., 2000; Robertson et al., 2009).

High-dose of ROS production is one of the significant properties of HY-PDT treatment of tumors (Agostinis et al., 2002). High ROS levels can reduce the amount of reduced intracellular GSH. Reduced GSH can combine with toxicants and be effluxed from the tumor cells to reduce the therapeutic efficacy of chemotherapy drugs, including paclitaxel (Asik et al., 2018). It was deduced that the decrease in intracellular reduced GSH levels after HY-PDT treatment could be used as a supplement to paclitaxel therapy. This may be one of the reasons why HY-PDT has an enhanced inhibitory effect on cancer cells after the addition of paclitaxel.

Paclitaxel and hypericin have both been reported in combination with other drugs for synergistic therapy in various types of cancers; for example, paclitaxel combined with metformin has been adopted as a new treatment strategy for APL^3^ (Lin et al., 2016a). In addition, hypericin combined with oxaliplatin has been shown to improve the therapeutic efficiency of colorectal cancer (Shukla and Gupta, 2008). The synergistic efficiency of HY-PDT with paclitaxel on HeLa cells was also reported by Wada et al. (2002), but the possible mechanism underlying the efficacy of the combined treatment is still unknown. The treatment of melanoma with a combination of drugs has not yet been performed. Therefore, the aim of this study was to evaluate the effects of paclitaxel combined with HY-PDT on cytokinetic and physiological parameters using the melanoma cell line B16-F10.

## Materials and Methods

### Cell Culture and MTT Analysis

B16-F10 cells were purchased from the Cell Bank of the Shanghai Institute of Cell Biology, Chinese Academy of Sciences (Shanghai, China).^2^ Cells were grown in RPMI 1,640 medium with phenol red, supplemented with 10% FBS, 100 U ml^−1^ penicillin, and streptomycin (Gibco Invitrogen Corp., Carlsbad, CA, United States), and incubated at 37°C in a humidified incubator containing 5% CO_2_. Cells were seeded in 96-well plates (2 × 10^4^ cells per well) or 6-well plates (1 × 10^5^ cells per well). After 12 h of incubation, paclitaxel and hypericin (Sigma-Aldrich Corporation, St. Louis, MO, United States) were added at different concentrations and incubated at 37°C for 24 h.

PDT was performed on B16-F10 cells, according to previously published guidelines.

Cells were seeded in 96-well plates (2 × 10^4^ cells/well) or 6-well plates (1 × 10^5^ cells/well). After settling down for 12 h, certain concentrations of paclitaxel and hypericin were added into the wells, which subsequently were kept in the dark at 37°C for 4 h. The cells including the control group were irradiated vertically for 1 h under 20 W/30 (Osram, Berlin, Germany) fluorescent tubes, and the irradiator fluence rate was adjusted to 3.15 mW cm^−2^, and then, the cells were transferred to normal culture conditions. The proliferation rate was assayed using the MTT method. The cells were treated with different concentrations of (0, 0.25, 0.5, 1, 2, and 4 μg/ml) paclitaxel or (0, 0.25, 0.5, 1, 2, and 4 μg/ml) HY or their combination, HY-PDT. After 24 h of treatment, 20 μl MTT (5 mg/ml in PBS) solution was added to each well and the cells were incubated for a further 4 h. The formed formazan crystals were dissolved in 150 μl of DMSO per well. The absorbance at 490 nm was measured using a microplate spectrophotometer (Bio-Rad, model 680). Cell viability was calculated as (A490 drug treated/A490 untreated control) × 100%. Each treatment was performed in triplicate, and each experiment was repeated thrice.

According to the results of the MTT assay, the optimal concentrations of paclitaxel and HY-PDT were 1 μg/ml when administered alone. We chose 0.5 μg/L paclitaxel and HY-PDT at a low concentration for our subsequent combination therapy.

### Nuclear Morphology Analysis by Fluorescent Microscopy

The cells were stained with Hoechst 33,342 (Beyotime Ins. Bio, China) at a final concentration of 2 μg/ml and incubated for 30 min in the dark at 37°C after drug treatment. Cells were photographed immediately under a fluorescence microscope (Nikon Corporation, Chiyoda-ku, Tokyo, Japan) with an excitation wavelength of 380 nm. Apoptotic cells were characterized by chromatin condensation and fragmentation. A minimum of 300 cells per treatment were evaluated.

### Analysis of Apoptosis-Related Protein Expression Levels

The expression levels of caspase-3, cleaved caspase-3, and PARP were detected by western blot analysis. Total proteins of the treated cells were extracted, and protein concentrations were evaluated using a BCA assay kit (Beyotime Ins. Bio, China). The required amount of protein was loaded onto SDS-PAGE gels for electrophoresis at 120 V, and the samples were transferred onto PVDF membranes using a VE186 transfer system (Tanon Ins. Bio, China) at 0.3 A for 90 min, which was activated by immersion in methanol for 5 s. The membrane was then washed with TBST and incubated overnight with 5% skim milk. The membrane was then incubated with primary antibodies against caspase-3, cleaved caspase-3, or PARP (Santa Cruz Biotechnology) for 2 h at room temperature. After washing three times in TBST, the membrane was incubated with secondary antibodies conjugated with horseradish peroxidase for 2 h at room temperature, washed three times again in TBST, and incubated with BeyoECL Plus (Beyotime Ins. Bio, China) for 5 min for the fluorescence intensity analysis. *β*-tubulin was used as the loading control.

### Intracellular ROS Level Assay

Cells were seeded in 6-well plates (1 × 10^5^ per well) and treated with drugs alone or in combination. ROS levels were evaluated using DCFH-DA^4^ oxidization methods (Beyotime Ins. Bio, China). Briefly, the culture medium was replaced with fresh serum-free medium containing 10 μM DCFH-DA after drug treatment and incubated for 30 min at 37°C. Intracellular ROS levels were evaluated by measuring the intensity of fluorescent DCF production from the fluorogenic substrate DCFH-DA. The fluorescence intensity of DCF was measured using a fluorescence microscope (Nikon Corporation, Chiyoda-ku, Tokyo, Japan).

### Mitochondrial Membrane Potential Detection

The rhodamine 123 staining method was used to measure the mitochondrial membrane potential. Rhodamine 123 (2-(6-amino-3-imino-3H-xanthen-9-yl) benzoic acid methyl ester) is a cationic yellowish-green fluorescent dye that selectively permeates biomembranes, including the mitochondria of living cells. It is widely used as a fluorescent probe for mitochondrial membrane potential and cell apoptosis detection. Cells were seeded in 6-well plates (1 × 10^5^ per well) and stained with rhodamine 123 (Beyotime Ins. Bio, China) at a final concentration of 2 μg/ml and incubated for 30 min in the dark at 37°C after incubation with single or combined drugs for 24 h. Fluorescence intensity was measured at 507 nm excitation and 529 nm emission under a fluorescence microscope (Nikon Corporation, Chiyoda-ku, Tokyo, Japan).

### Intracellular GSH, GSSG Level, and GR Activity Assay

The levels of GR activity, reduced glutathione (GSH), and oxidized glutathione (GSSG) were detected using assay kits (Beyotime Ins. Bio, China) after the drug treatment.

Glutathione reductase catalyzes the substrate of oxidant glutathione (GSSG) to produce reduced glutathione (GSH) with NADPH. GSH reacts with its chromogenic substrate DTNB to form the yellow substance TNB. The level of TNB was determined by measuring the absorbance at 412 nm using a microplate spectrophotometer (Bio Rad, model 680).

### Biostatistical Analysis

All experiments were repeated at least three times. Fluorescence intensity was analyzed using ImageJ software (V8.0). All data expressed as the mean value ± standard deviation (SD) from a representative experiment, followed by the Kruskal–Wallis test for multiple comparisons (*p* values < 0.05), were considered statistically significant.

## Results

### Cytotoxicity of Paclitaxel and HY on B16-F10 Cells

The IC_50_ of hypericin was above 4 μg/ml, that of HY-PDT was around 1.8 μg/ml, and the IC_50_ of paclitaxel alone after treatment for 24 h was approximately 2.6 μg/ml. However, combined paclitaxel with HY-PDT showed more intensive cytotoxicity in B16-F10 cells, with an IC_50_ of about 0.5 μg/ml for each compound. The IC_50_ was about 2 μg/ml for each compound if hypericin was not photoactivated. Compared with the photoactivated HY combined with paclitaxel group, the cytotoxicity was much lower.

**FIGURE 1:**
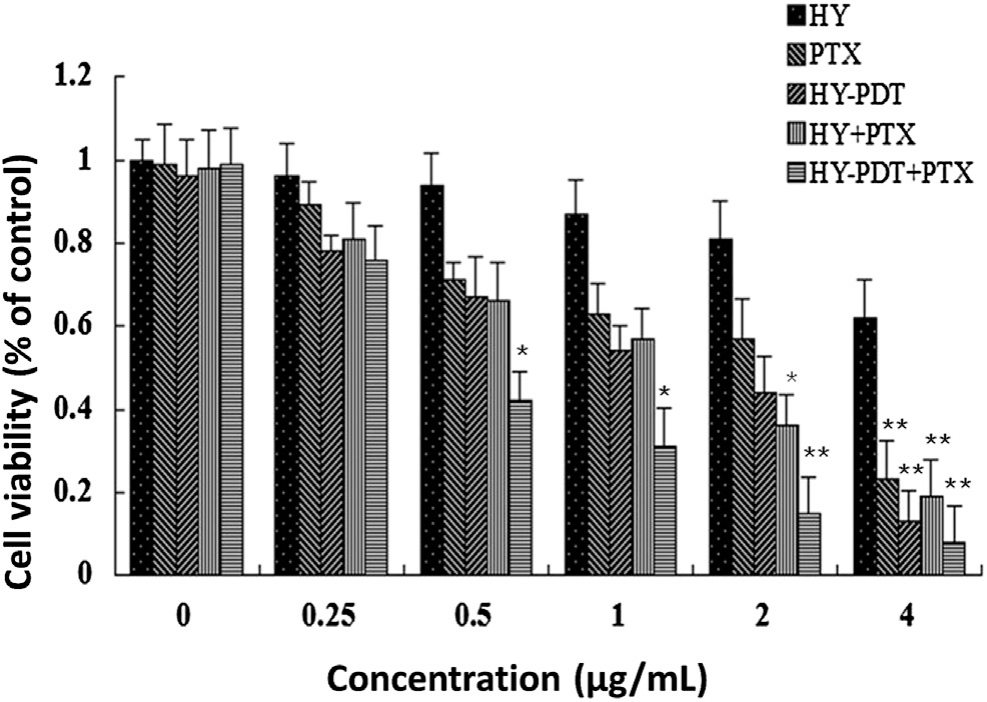
Cytotoxicity of different doses of paclitaxel and HY-PDT on B16-F10 cells. The proliferation rate was assayed by the MTT method. The cells were treated with different concentrations (0, 0.25, 0.5, 1, 2, and 4 μg/ml) of paclitaxel or (0, 0.25, 0.5, 1, 2, and 4 µg/ml) HY or their combination, HY-PDT treatment. All data are expressed as mean ± SEM from three independent experiments. For the HY-PDT treatment, cells were irradiated vertically for 1 h under a 20 W/30 quartz–halogen lamp (Osram, Berlin, Germany) after hypericin was added, the irradiator fluence rate was adjusted to 3.15 J/cm^2^, and the cells were then transferred back into normal culture conditions and combined with the corresponding concentrations of paclitaxel. *p < 0.05, **p < 0.01, compared to the control.

As Figure 1 shows, if HY was not photoactivated, the IC_50_ value for the combination of paclitaxel and HY was 2 μg/ml. This was close to the IC_50_ values for HY and paclitaxel alone. The combination of paclitaxel and HY showed no significant synergistic effect on cytotoxicity if hypericin was not photoactivated. Therefore, we did not continue to use a combination of paclitaxel and nonphotoactivated HY in the following mechanistic experiments.

After 24 h exposure under 0.5 μg/ml paclitaxel and HY-PDT, the inhibition rate was 42% of that of the controls. As shown in Figure 1, the two drugs showed synergistic effects. In subsequent experiments, the concentrations of paclitaxel and HY-PDT were 1 μg/ml when administered alone. We chose 0.5 μg/L paclitaxel and HY-PDT at the low concentration for our subsequent combination therapy.

### Apoptosis Analysis of B16-F10 Cells After Treatment With Paclitaxel and HY-PDT

**FIGURE 2:**
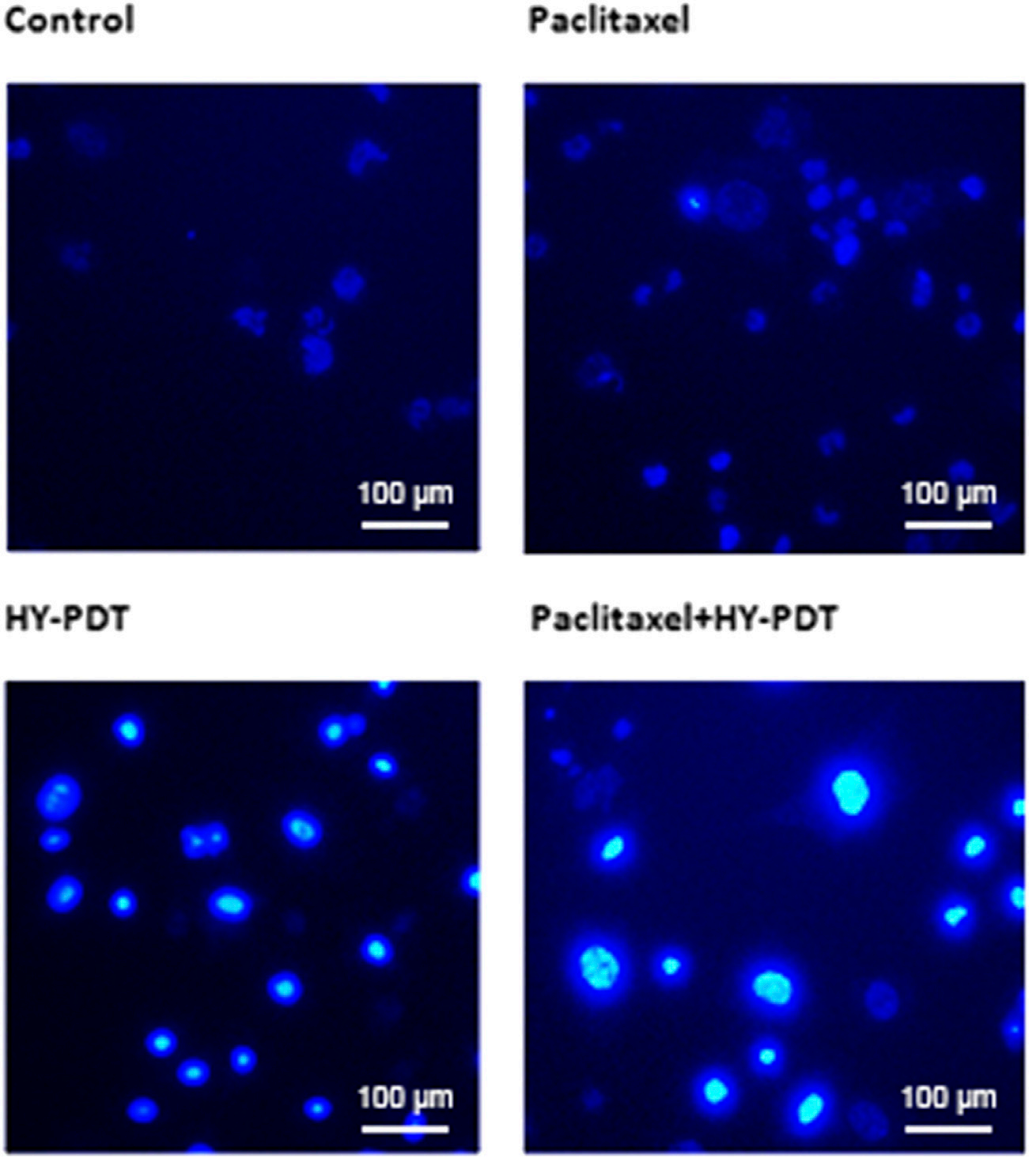
Morphology detection of B16-F10 nucleus after treatment with paclitaxel or HY-PDT or the combination. Fluorescence graph of Hoechst 33,342 staining after B16-F10 cells were treated with paclitaxel (1 μg/ml) and HY-PDT (1 μg/ml) alone or in combination (0.5 μg/ml each). Scale bars: 100 μm.

The inhibitory effect of paclitaxel combined with HY-PDT on B16-F10 cells was accompanied by an increase in apoptosis. Apoptosis was confirmed by nuclear morphology analysis using membrane-permeable Hoechst 33,342 fluorescent stain. Nuclear morphological changes, such as chromatin fragmentation, bi- and/or multinucleation, and dot-like chromatin condensation (an indicator of late apoptosis), were observed more frequently in the (0.5 μg/ml + 0.5 μg/ml) combined group, as shown in Figure 2. These results provide further evidence for apoptosis induction by the combined drugs, and the results showed the potential of this drug combination as an efficient anticancer therapy, since the apoptosis-inducing ability is critical to determining the efficacy of anticancer drugs.

### Mitochondrial Membrane Potential Analysis After Drug Treatment

**FIGURE 3:**
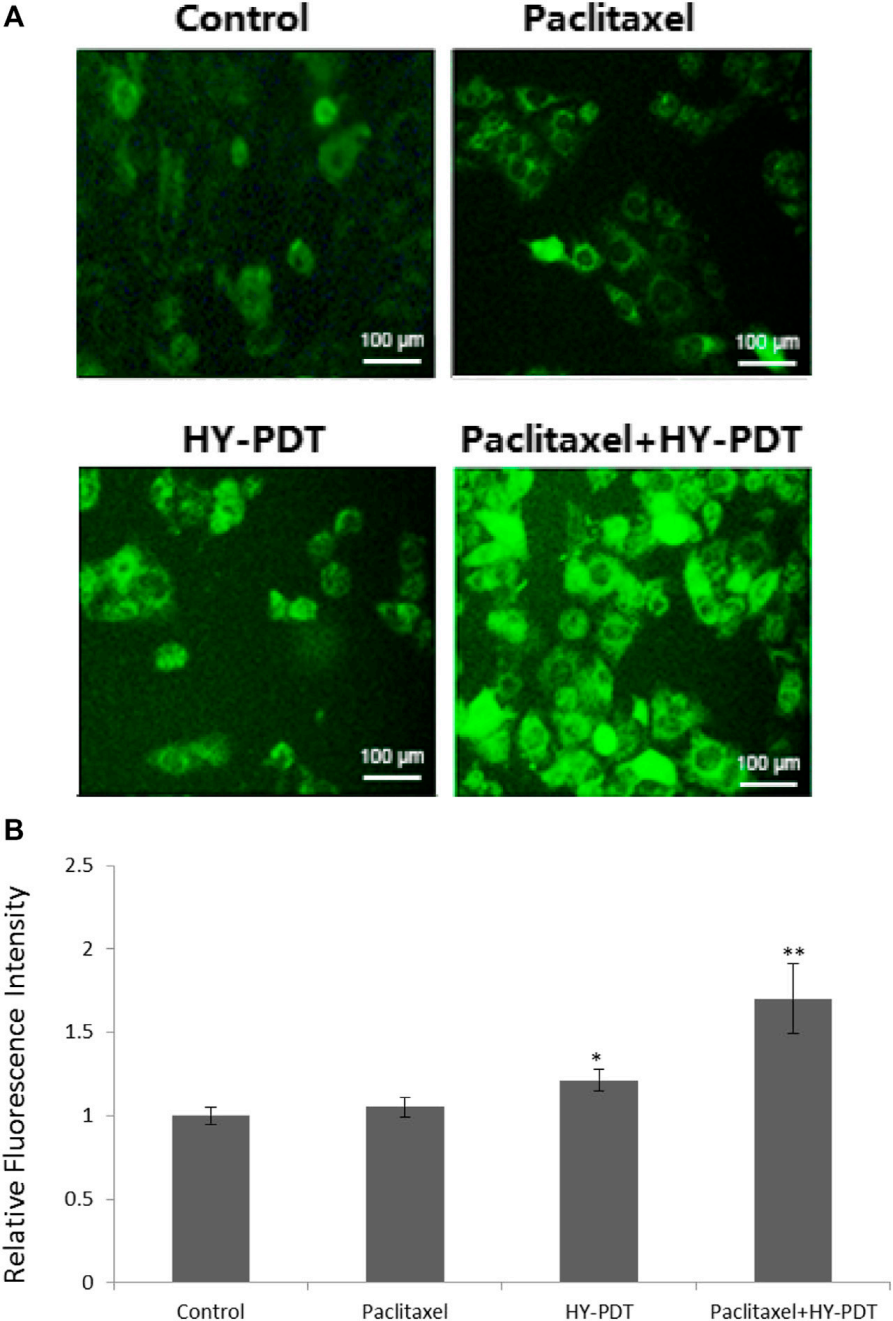
Mitochondrial membrane potential detection in B16-F10 cells. **(A)** Fluorescence microscope detection of B16-F10 cells treated with paclitaxel (1 μg/ml) and HY-PDT (1 μg/ml) alone or in combination (0.5 μg/ml each). Scale bars: 100 μm. **(B)** ImageJ analysis of relative intensity. *p < 0.05, **p < 0.01, compared to the control.

The intracellular fluorescence intensity of rhodamine 123 remained unchanged after treatment with 1 μg/ml paclitaxel; however, it was 1.18-fold higher than that of the control after 1 μg/ml HY-PDT-treatment and was 1.7-fold higher than that of controls after combining 0.5 μg/ml paclitaxel with 0.5 μg/ml HY-PDT (Figure 3).

The results indicated that paclitaxel combined with HY-PDT could decrease the mitochondrial membrane potential, and the results showed the collapse of mitochondria after treatment with the two drugs.

### Analysis of Apoptosis-Related Proteins After Drug Treatment

**FIGURE 4:**
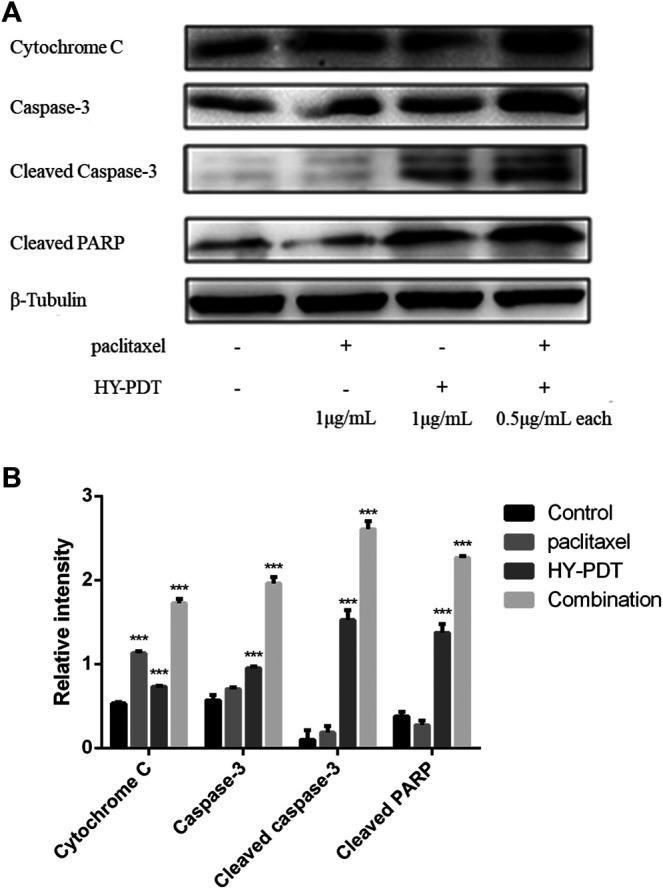
Effect of paclitaxel and HY-PDT on the expression of apoptosis regulators. **(A)** The levels of cytochrome c, caspase-3, and PARP were measured by western blotting analysis after paclitaxel (1 μg/ml), HY-PDT (1 μg/ml), and the combined (0.5 μg/ml each) treatment. Cytochrome C, 15 kDa; caspase-3, 32 kDa; cleaved caspase-3, 17, and 19 kDa; and cleaved PARP, 89 kDa. The original western-blot images are in the Supplementary Figures. **(B)** ImageJ analysis of relative intensity, ***p < 0.001, compared to the control.

As shown in Figure 4, the concentration of cytochrome *c* increased in the HY-PTD combination group. Paclitaxel combined with HY-PDT (each 0.5 g/ml) was 112% higher than that of HY-PDT (1 g/ml) alone and 158% higher than that of paclitaxel (1 g/ml) alone. A high level of cytochrome *c* is considered a typical characteristic of apoptosis.

This increase of cytochrome *c* induced the expression of caspase-3, resulting in cleavage of the DNA repair protein PARP (Lin et al., 2016b). As shown in Figure 4, the expression levels of caspase-3 and cleaved PARP in the combination group were 240 and 160% of those in the control group, respectively, and increased cytochrome *c* levels were related to mitochondrial membrane collapse and ROS-induced mitochondrial damage. The increased expression levels of cleaved caspase-3 and PARP indicated that the caspase–protein-dependent pathway was involved in B16-F10 cell apoptosis induced by the drug combination. The results indicated that the potential mechanism of paclitaxel combined with HY-PDT to promote B16-F10 cell apoptosis might be related to the mitochondrial damage pathway.

### Paclitaxel Combined With HY-PDT Reduced GSH Level and GR Activity

**FIGURE 5:**
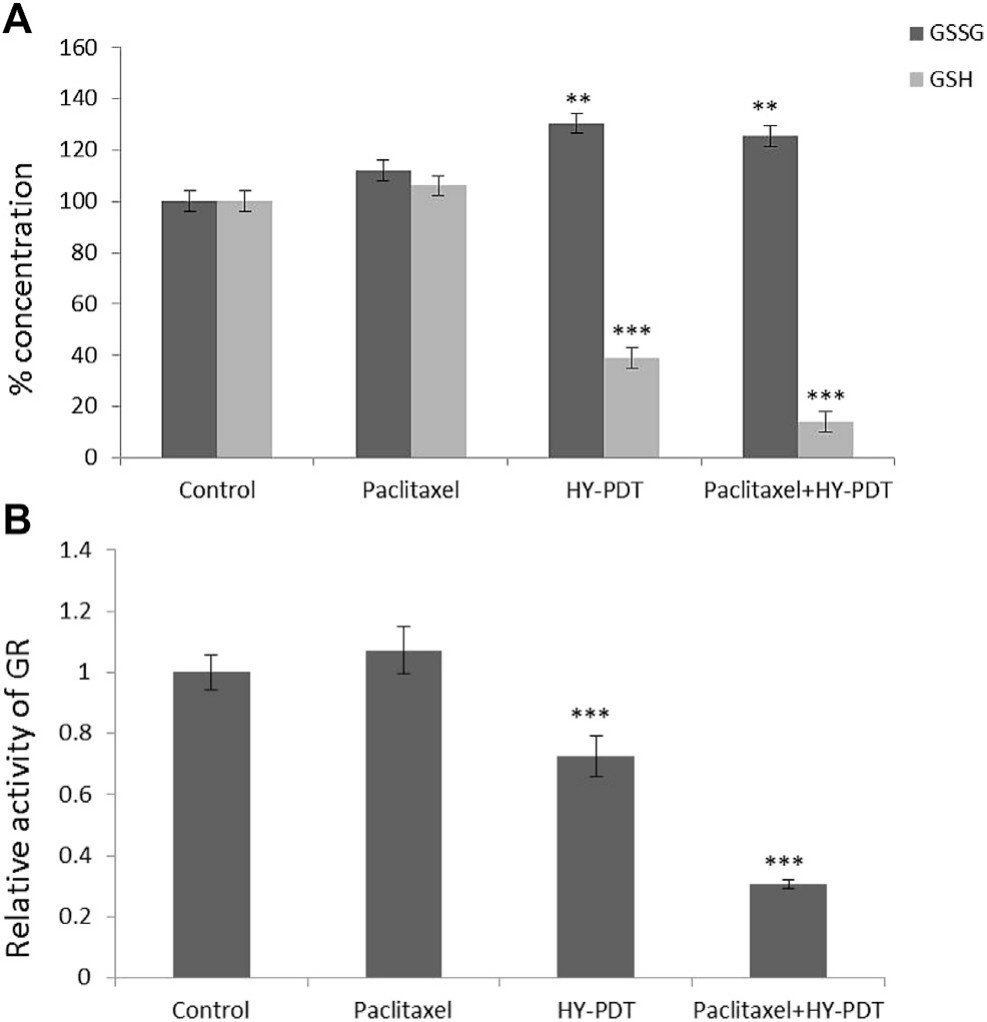
Analysis of GSH/GSSG levels and relative activity of GR in B16-F10 cells. The cells were treated with the paclitaxel (1 μg/ml) and HY-PDT (1 μg/ml) alone or in combination (0.5 μg/ml each). **(A)** Intracellular concentration of GSSG and GSH. **p < 0.01, ***p < 0.001, compared to control. **(B)** Relative activity of GR. ***p < 0.001, compared to control.

Glutathione (GSH) plays a vital role in the detoxification of paclitaxel (Brown et al., 2004). The amount of GSH was reduced by 61% after 1 μg/ml HY-PDT treatment, while the ratio of GSSG/GSH increased by approximately 30%. Moreover, a more severe decline in GSH concentration was observed after treatment with 0.5 μg/ml paclitaxel combined with 0.5 μg/ml HY-PDT, which was approximately 86% lower than that in the control cells (Figure 5A).

Considering the predictions of the online molecular interaction simulation platform of TarPred (http://202.127.19.97:5555/create, Table 1), glutathione reductase (GR) was proposed as a possible target for hypericin. After combination treatment, GR activity was downregulated, followed by decreased intracellular GSH levels (Figure 5B).

**TABLE 1:** The ten most likely targets of hypericin predicted by the TarPred platform. Higher scores indicate a higher likelihood.

	Targets	Scores
1	Glutathione reductase	0.637
2	Cytochrome P450 3A43(P450 3A43)	0.483
3	Corticotropin-releasing factor receptor 1	0.457
4	Dopamine D3 receptor	0.453
5	Estrogen receptor alpha	0.34
6	Thioredoxin reductase 2	0.33
7	Monoamine oxidase B	0.313
8	CDK5	0.287
9	Aromatase (CYP19) (CYP19)	0.283
10	PIM-1 kinase(PIM-1)	0.283

**FIGURE 6:**
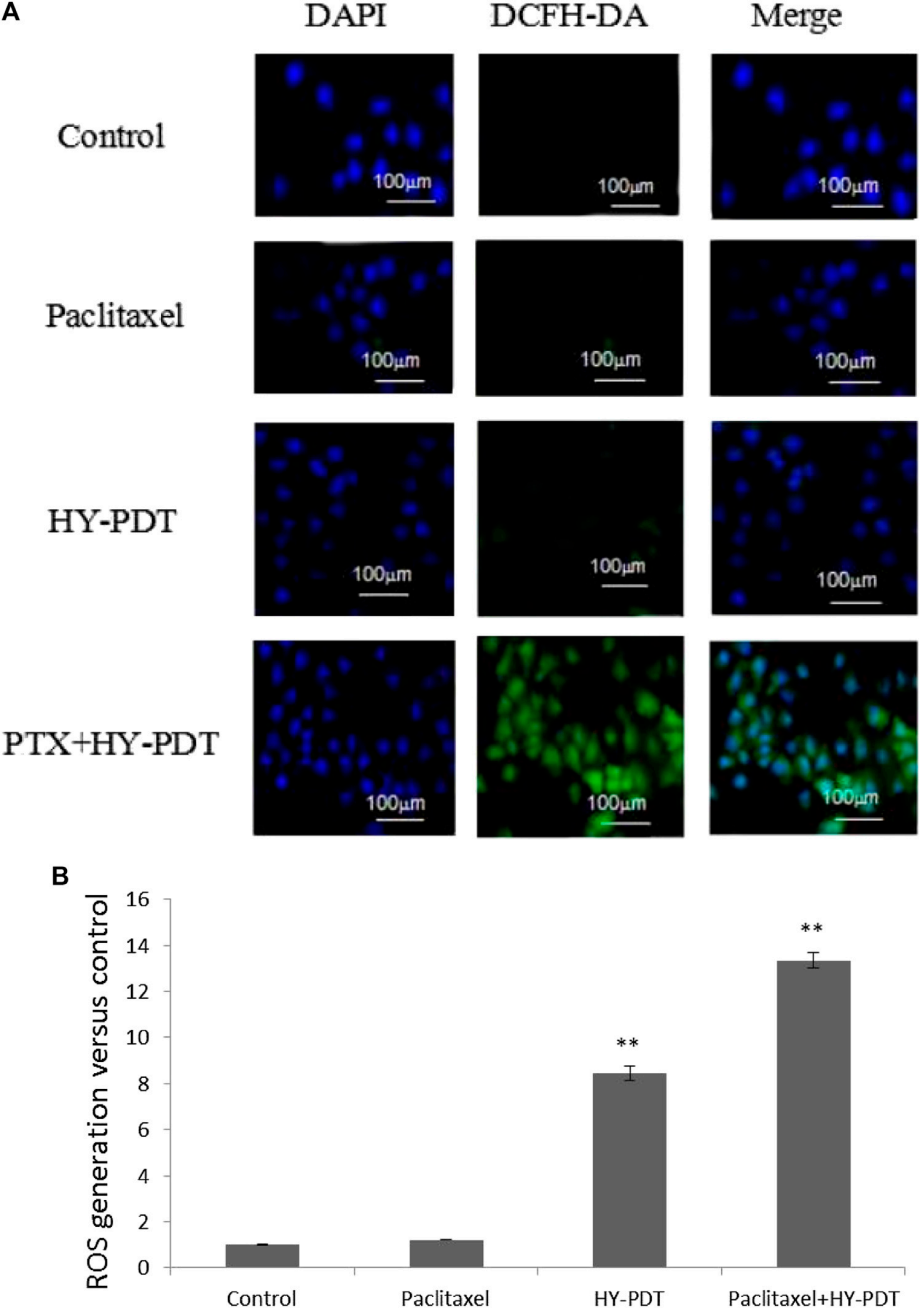
ROS level analysis of B16-F10 cells by fluorescence intensity after drug treatment. **(A)** B16-F10 cells were treated with the paclitaxel (1 μg/ml) and HY-PDT (1 μg/ml) alone or in combination (0.5 μg/ml each). Generation of ROS was observed under a fluorescence microscope. Scale bars: 100 μm. **(B)** ImageJ processing calculation to obtain relative fluorescence intensity. **p < 0.01, compared to control.

ROS generation was observed using a fluorescence microscope. ImageJ processing calculation was performed to obtain the relative fluorescence intensity. **p < 0.01. The intracellular ROS level detection results showed that the ROS level in the combination group was significantly increased, at 12.6 times higher than that in the control group, and the ROS level in the HY-PDT treatment group was only 8.2 times higher than that in the control group (Figures 6A,B).

## Discussion

ROS have been reported to play an important role in drug-induced apoptosis. The production of ROS is accompanied by inactivation of superoxide dismutase (SOD), cytochrome *c* release, caspase-3 activation, and PARP cleavage, leading to apoptosis of numerous types of cancer cells (Lin et al., 2016b; Li et al., 2020). High levels of ROS are accompanied by a decrease in GSH levels. It has been reported that GSH can stimulate the efflux of anticancer drugs, such as the chemotherapy drug paclitaxel (Schuitmaker et al., 1996). It can, therefore, be hypothesized that reducing intracellular GSH concentration might be the best choice to overcome GSH-induced drug resistance and improve the therapeutic effect of chemotherapy drugs.

We studied the synergistic effect of paclitaxel and HY-PDT on melanoma cells and found that HY-PDT induced ROS more significantly and enhanced the inhibitory effect of paclitaxel at a low dose (0.5 μg/ml each). The ROS level increased 12.6-fold in the combination group compared to the control group and was higher than that in the HY-PDT-treated group, which was approximately 8.2-fold higher than that in the control group (Figure 6). The combined treated cells had lower GSH levels and GR activity than HY-PDT-alone–treated cells; they were 36 and 71% lower than that of the control group, respectively. It has previously been reported that, under low GSH levels and GR activity, drug-related transport proteins will be blocked and chemotherapy drugs, such as paclitaxel, accumulate more easily in cells (Šemeláková et al., 2016). In addition, it has been reported that HY-PDT can inhibit the growth of tumor cells by reducing the level of growth differentiation factor 15 (GDF-15) and disturbs the surviving inhibitor YM155, which is also sensitive to paclitaxel (Kuchárová et al., 2015; Gyurászová et al., 2016). The results of our research showed that B16-F10 cells treated with HY-PDT were more sensitive to paclitaxel and underwent a synergistic effect in promoting cell apoptosis.

The results showed that the ratio of reduced glutathione (GSH) to oxidized glutathione (GSSG) was significantly decreased and glutathione reductase (GR) was significantly inactivated after the combination treatment, which indicated that the drug combination could reduce the level of reduced glutathione by inhibiting glutathione reductase, thereby inhibiting the cell resistance to oxidative stress (Chawla et al., 2018).

**FIGURE 7:**
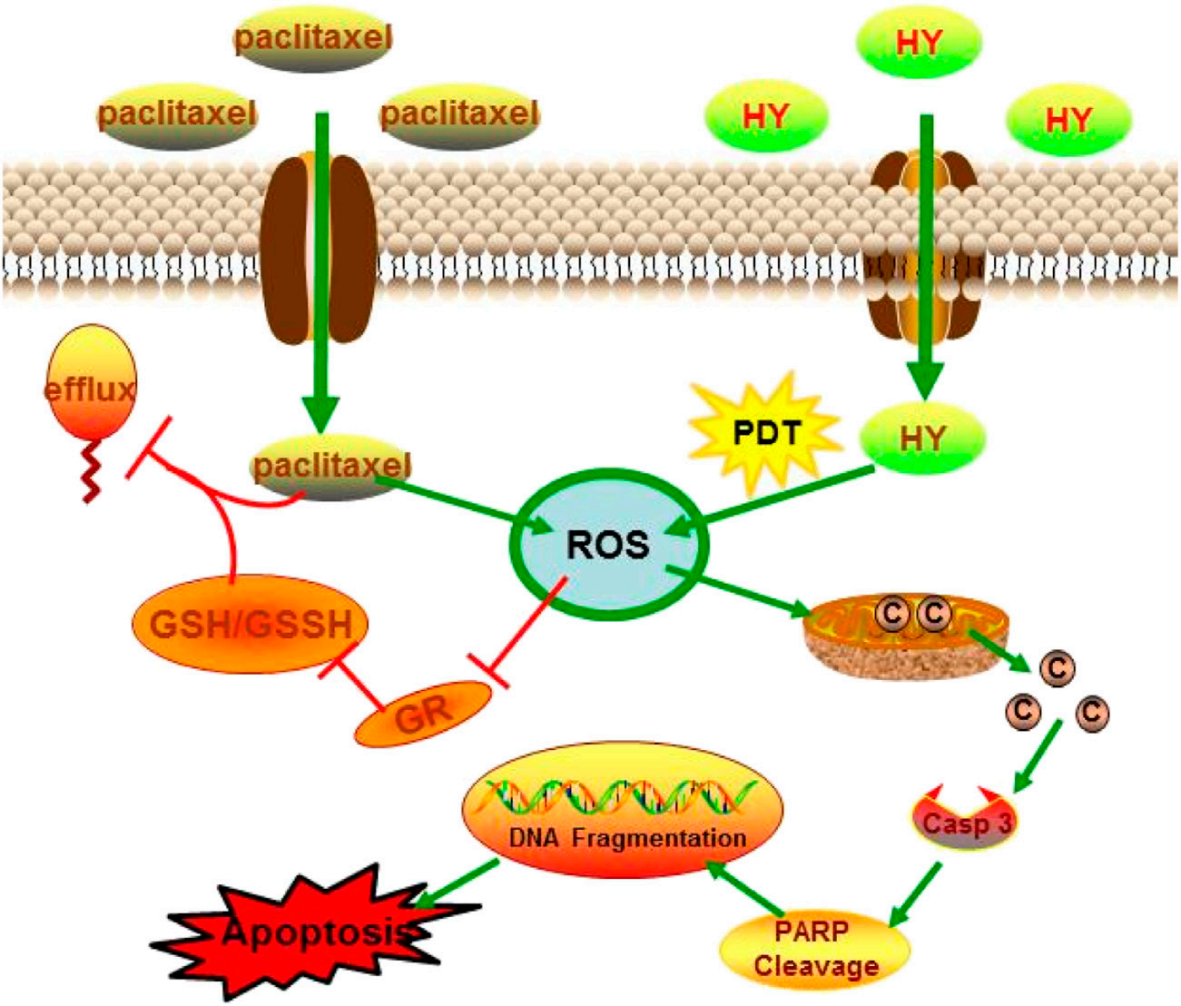
Schedule of HY-PDT combined with paclitaxel enhanced apoptosis in B16-F10.

It has been reported that paclitaxel can form complexes with GSH to be transported out of cells. HY-PDT decreases intracellular GSH levels by stimulating ROS production after irradiation and led to the accumulation of paclitaxel in B16-F10 cells, as shown in Figure 7. The synergistic effect of increased ROS levels and intracellular accumulation of paclitaxel may promote the apoptosis of B16-F10 cells. Caspase-3 is an important protein that is usually used to detect cell apoptosis (Meloni et al., 2003). The mechanism of apoptosis induced by HY-PDT in HeLa cells is mediated by the intrinsic cell death pathway, which involves the release of mitochondrial cytochrome *c,* followed by the activation of caspase-3 expression (Vantieghem, 2001), because the HY-PDT-induced apoptosis of HeLa cells can be reduced by a caspase-3 inhibitor (Devarajan et al., 2002). The release of cytochrome *c* from the mitochondria into the cytosol can also induce the activation of procaspase-9, which is considered as the initial component of procaspase-3 protein. Activated caspase-9 stimulates the activity of procaspase-3 and apoptosis (Vantieghem et al., 1998). Procaspase-3 is cleaved to activate cleaved caspase-3;upregulation of cleaved caspase-3 was observed in HY-PDT- and paclitaxel-treated B16-F10 cells, at levels 1.9- and 11.7-fold higher than those of the control group, respectively. The expression level of the combined group was further enhanced, at 26.6-fold higher than that of the control group (Figure 4). Our study demonstrates the potential mechanism of HY-PDT combined with paclitaxel in melanoma treatment.

## Conclusion

This study explored the possible mechanism underlying enhanced apoptosis induced by paclitaxel combined with HY-PDT on mouse melanoma B16-F10 cells. Photoactivated hypericin combined with paclitaxel produces high levels of cytotoxic ROS. High ROS levels further reduced the mitochondrial membrane potential and induced mitochondrial membrane collapse, followed by the release of cytochrome *c* into the cytoplasm. Higher cytochrome *c* levels activated the expression of cleaved caspase-3 and cleaved PARP, both of which are apoptosis factors, thus inducing DNA damage and apoptosis in B16-F10 cells. Moreover, higher ROS also inhibited the activity of GR and reduced the ratio of GSH/GSSG, while reduced GSH content could increase the intracellular accumulation of paclitaxel. According to the results of our study, HY-PDT combined with paclitaxel improved the apoptotic efficiency of B16-F10 cells.

In conclusion, the enhanced apoptosis of cells treated with a combination of paclitaxel and HY-PDT on B16-F10 cells is a potential option for developing new treatment strategies for melanoma therapy.

## Data Availability

The original contributions presented in this study are included in the article/Supplementary Material; further inquiries can be directed to the corresponding author.

## Footnotes

2 MTT: 3-(4,5-dimethylthiazol-2-yl)-2,5-diphenyltetrazolium bromide.

3 APL: acute promyelocytic leukemia.

4 DCFH-DA: 2,7-dichlorodihydrofluorescein diacetate.
