# Supplementary figures and images for: Hypericin Enhances Paclitaxel-Induced B16-F10 Cell Apoptosis by Activating a Cytochrome c Release–Dependent Pathway
Source: Front Pharmacol. 2021 Aug 4;12:652452. doi: 10.3389/fphar.2021.652452 (PMC8371448; doi:10.3389/fphar.2021.652452)

Fig.4

Cytochrome C


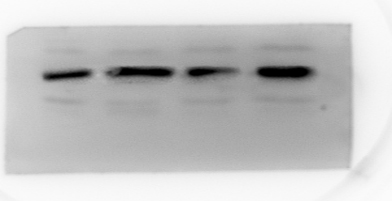


Caspase-3


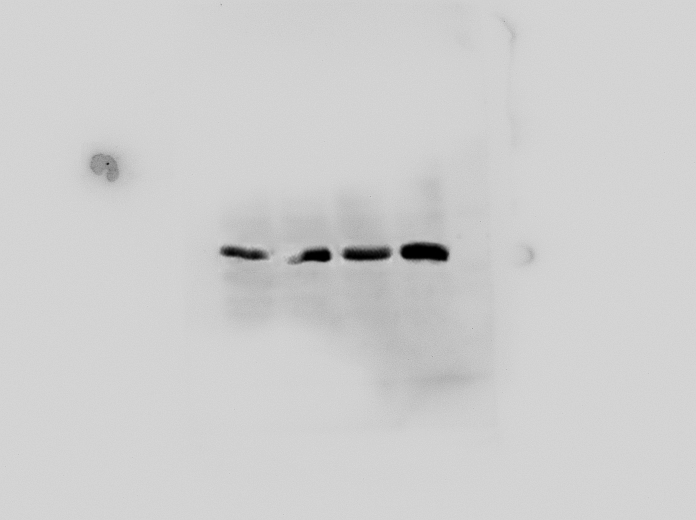


Cleaved PARP


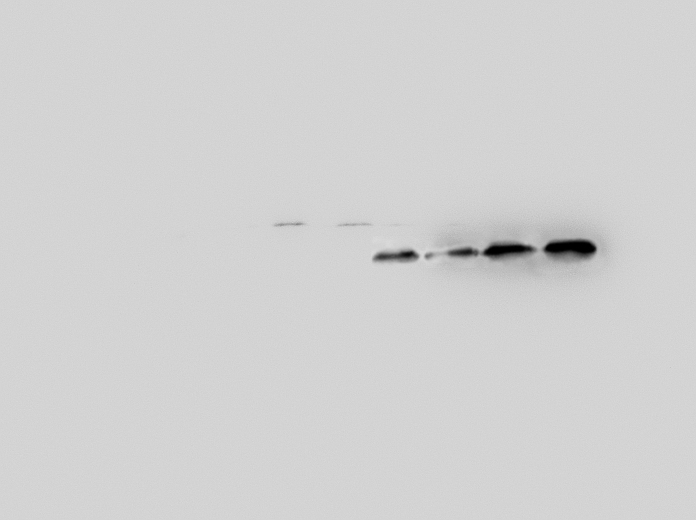


Β-Tubulin


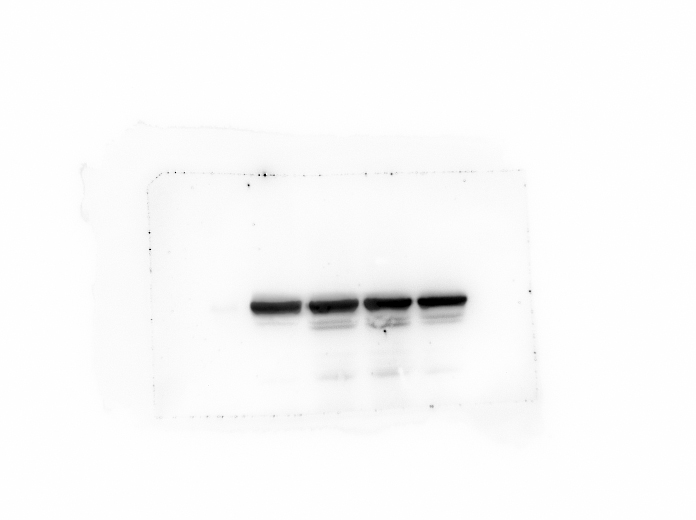

Supplement: Supplementary file 1 [file DataSheet1.DOCX]
